# Genomic and enzymatic evidence of acetogenesis by anaerobic methanotrophic archaea

**DOI:** 10.1038/s41467-020-17860-8

**Published:** 2020-08-07

**Authors:** Shanshan Yang, Yongxin Lv, Xipeng Liu, Yinzhao Wang, Qilian Fan, Zhifeng Yang, Nico Boon, Fengping Wang, Xiang Xiao, Yu Zhang

**Affiliations:** 1 School of Oceanography, Shanghai Jiao Tong University, Shanghai, P. R. China; 2 State Key Laboratory of Ocean Engineering, Shanghai Jiao Tong University, Shanghai, P. R. China; 3 School of Life Sciences and Biotechnology, Shanghai Jiao Tong University, Shanghai, P. R. China; 4 Center for Microbial Ecology and Technology, Ghent University, Ghent, Belgium; 5 Laboratory for Marine Biology and Biotechnology, Pilot National Laboratory for Marine Science and Technology, Qingdao, Shandong P. R. China

**Keywords:** Microbial ecology, Carbon cycle, Marine biology

## Abstract

Anaerobic oxidation of methane (AOM) mediated by anaerobic methanotrophic archaea (ANME) is the primary process that provides energy to cold seep ecosystems by converting methane into inorganic carbon. Notably, cold seep ecosystems are dominated by highly divergent heterotrophic microorganisms. The role of the AOM process in supporting heterotrophic population remains unknown. We investigate the acetogenic capacity of ANME-2a in a simulated cold seep ecosystem using high-pressure biotechnology, where both AOM activity and acetate production are detected. The production of acetate from methane is confirmed by isotope-labeling experiments. A complete archaeal acetogenesis pathway is identified in the ANME-2a genome, and apparent acetogenic activity of the key enzymes ADP-forming acetate-CoA ligase and acetyl-CoA synthetase is demonstrated. Here, we propose a modified model of carbon cycling in cold seeps: during AOM process, methane can be converted into organic carbon, such as acetate, which further fuels the heterotrophic community in the ecosystem.

## Abstract (web summary)

Ocean cold seeps are poorly understood relative to related systems like hydrothermal vents. Here the authors use high pressure bioreactors and microbial communities from a cold seep mud volcano and find a previously missing step of methane conversion to acetate that likely fuels heterotrophic communities.

## Introduction

Cold seeps are areas, where hydrocarbon-rich fluid seeps up from below the ocean floor at fluid-flow velocities of centimeters to meters per year, often as hydrogen sulfide and methane^1,2^. They are common along continental margins worldwide and can be thought of as hot spots of a certain habitat type, providing niches that are strongly different from the surrounding seafloor^3–5^. The chemosynthetic microorganisms inhabiting cold seeps convert the methane into organic matter and carbon dioxide to generate energy. Anaerobic oxidation of methane (AOM) coupled to sulfate reduction (SR) is the primary energetic process in cold seeps and is catalyzed by a consortium of anaerobic methanotrophic archaea (ANME) and sulfate-reducing bacteria (SRB) of the Deltaproteobacteria^6–8^. The overall AOM-SR reaction CH_4_ + SO_4_^2−^ → HS^−^ + HCO_3_^−^ + H_2_O generates a Gibbs free energy of only −20 to −40 kJ/mol of methane oxidized, which is shared between ANME and SRB. Therefore, AOM is considered one of the least exergonic processes supporting life^9^. Meanwhile, the methane-fueled anoxic sediments above gas vents and gas hydrates are one of the most populated marine microbial ecosystems, reaching 10^7^–10^9^ cells/cm^3^^10,11^. In addition, a large number of metazoans (such as tubeworms, bivalves, etc.) depend on the energy flow through microbial processes^12^. Moreover, the AOM process is also a major sink of the oceanic methane budget, consuming up to 300 Tg methane per year, equivalent to ~88% of the methane released from subsurface reservoirs^13^. Hence, the AOM process attenuates the emission of the greenhouse gas methane and supports a large, diverse microbial, and animal population.

In addition to ANME and SRB, large heterotrophic bacterial populations exist in cold seeps. For example, 58.3% of candidate phylum Atribacteria (formerly known as JS1) are heterotrophic anaerobes and can catabolize organic acids such as acetate and propionate via the methylmalonyl-CoA pathway in the sulfate methane transition zone of Ulleung Basin^14,15^. Two cold seeps in the South China Sea harbor dominant populations of Firmicutes, Chloroflexi, Actinobacteria, Atribacteria, and Bacteroidetes, in which the heterotrophic bacteria represent up to 70% of the total biomass^16^. This is surprising because the primary energy source is the AOM process, where carbon dioxide is the end product. To support a large and stable heterotrophic community, a sustainable source of organic carbon is required but has not been discovered. Therefore, we put forward our research questions: is it possible to produce organic carbon during the AOM process, thus further fueling the heterotrophic community in cold seep ecosystems? And if so, what could be the underlying mechanism? Early studies had already suggested that ANME-2 is closely related to the acetotrophic methanogen *Methanosarcina* and performs a reversal of H_2_-independent methanogenesis^17^. Moreover, when the methyl-coenzyme M reductase (Mcr) from ANME-1 is cloned into the methanogen *Methanosarcina acetivorans*, *M. acetivorans* can oxidize methane to produce acetate^18^. Therefore, we propose that acetate may be produced by ANME and serve as a carbon source to support the large heterotrophic community. As demonstrated by a previous study, elevated partial pressures of CH_4_ increased the gained Gibbs free energy and thus stimulated in vitro SR-AOM activity^19^. Herein, we address the above questions by using a continuous high-pressure bioreactor system to simulate the cold seep environment and create a simplified ecosystem supported only by the AOM process. When methane and sulfate are supplied as the only energy sources in the ecosystem for 8 months, both AOM activity and acetate production are detected. Furthermore, the acetogenesis by ANME is confirmed by genomic and enzymatic evidence. Therefore, we propose a conceptual model of carbon cycling in cold seeps, where acetate release from AOM process is considered.

## Results

### A microbial community fueled solely by the AOM process

**Fig. 1:**
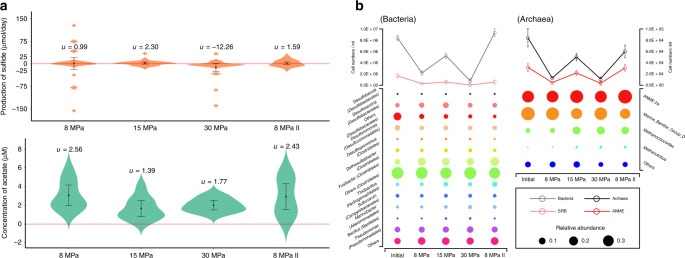
Activity and community structure in the enrichment. The mean value (*u*) and standard deviation of sulfide production are calculated based on: *n* = 27 (8 MPa), *n* = 28 (15 MPa), *n* = 25 (30 MPa), *n* = 28 (8 MPa II). The mean value (*u*) and standard deviation of acetate concentration are calculated based on: *n* = 10 (8 MPa), *n* = 10 (15 MPa), *n* = 9 (30 MPa), *n* = 10 (8 MPa II). The mean value and standard deviation of cell numbers are calculated based on *n* = 3.

An AOM-enriched community, which originated from a mud volcano in Gulf of Cadiz, was incubated in a simulated cold seep ecosystem where methane and sulfate were supplied as the only energy source within the ecosystem for 8 years prior to this research^19^. The incubation was performed in a continuous high-pressure reactor with independent control of the methane partial pressure and incubation pressure^19^. To test the stability of this AOM-enriched community, the incubation pressure was changed every two months: 8, 15, 30, and 8 MPa (II). The detailed incubation conditions are described in the “Methods” section. The chemical composition in the incubation system was measured every two days to track AOM activity (Supplementary Fig. 1). Approximately 0.99–2.30 μmol sulfide production per day was observed in all the tested conditions except the one at 30 MPa, when the community was likely disturbed by such high pressure and a negative average sulfide production was observed (−12.26 μmol sulfide production per day) (Fig. 1a). Moreover, throughout the incubation period, approximately 1.39–2.56 μM acetate was detected in the slurry, and the highest acetate accumulation was observed under 8 MPa methane and 8 MPa incubation pressure (Fig. 1a). The microbial conversion of methane into acetate was further confirmed by isotope-labeling experiments. This AOM-enriched community was subsampled and incubated with 85 mM dissolved ^13^C–CH_4_, which is the calculated methane saturation concentration under 8 MPa methane partial pressure, and 30 mM dissolved ^12^C–HCO_3_^−^ as the carbon sources. Under 8 or 30 MPa incubation pressure, the sulfide concentration increased from 9.15 μM to 92.01 or 15.75 μM respectively, the ^13^C–HCO_3_^−^ abundance among total HCO_3_^−^ in the liquid phase increased from natural abundance of 1.08% to 1.31%, the final concentration of ^13^C-acetate (with molecular weight of 61) reached to 0.45 or 0.32 μM, respectively (Table 1). ^13^C-acetate with molecular weight of 62 was not detected. Therefore, it is likely that ^13^C-acetate was mainly converted from ^13^C-CH_4_ directly, rather than from ^13^C–CO_2_ through acetogenesis.

**Table 1:** Sulfide, ^13^C-acetate concentrations and ^13^C–CO_2_ abundance after incubation in the fed-batch experiment.

Dissolved ^13^C–CH_4_ concentration (mM)	Incubation pressure (MPa)	Sulfide concentration after incubation (μM)^a^	^13^C–CO_2_ abundance among total CO_2_ after incubation^a^	^13^C-acetate concentration after incubation (μM)^a^
85	8	92.01 ± 5.82	1.31% ± 0.00%	0.45 ± 0.27
85	30	15.75 ± 3.72	1.31% ± 0.01%	0.32 ± 0.03

^a^Data are mean values with standard deviations from triplicate incubations.

The microbial communities of each incubation stage were analyzed based on the 16S rRNA gene sequence data. In archaeal community, ANME-2a and Marine_Benthic_Group_D (MBG-D) were the main groups, which contribute 36.3–52.3% and 27.7–49.3%, respectively (Fig. 1b). On the other hand, the bacterial community was relatively more diverse than archaeal community; SRB accounted for less than 11% with Desulfosarcina (3.9–7.3%), Desulfobacula (0.1–0.2%), SEEP–SRB1 (0.1–0.2%), and uncultured Desulfobacteraceae (2.2–4.9%), while the dominant groups were Clostridiales (55.0–59.8%), Pseudomonadales (6.8–8.8%), Oceanospirillales (3.0–7.0%), Desulfuromonadales (3.7–5.6%), and Hydrogenophilales (2.3–5.0%) (Fig. 1b). The ANME and SRB cells ranged from 4.2 × 10^3^ to 3.1 × 10^4^ cells/mL and 1.3 × 10^4^ to 1.4 × 10^5^ cells/mL, respectively (Fig. 1b).

### Genomic evidence of methane to acetate conversion by ANME-2a

**Fig. 2:**
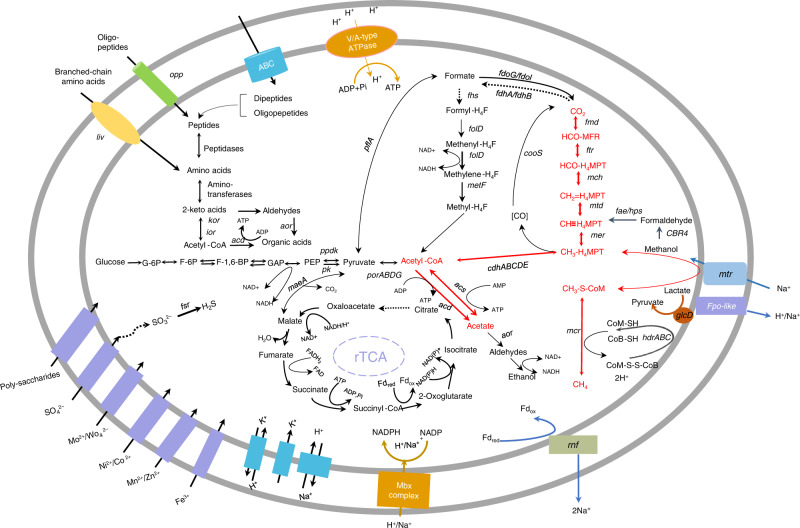
Reconstruction of ANME-2a metabolic pathways. The solid arrows indicate the presence of genes and the dashed arrows indicate the absence of genes involved in the processes based on metagenome-assembled genome.

To verify whether ANME-2a has the metabolic potential to convert methane into acetate and to identify the key genes involved, a metagenomic approach was applied. DNA was extracted from the biomass after each incubation stage. A total of 126,043,665 reads passed the quality control criteria. De novo assembly of the metagenomic reads and binning by tetranucleotide signatures revealed a total of five metagenome-assembled genomes (MAGs) belonging to ANME with high quality (Supplementary Table 1). The taxonomic identity of each MAG was verified by the construction of a phylogenetic tree based on whole-genome information (Supplementary Fig. 2). The details of the data processing are described in the “Methods” section. A complete metabolic pathway converting methane to acetate was identified in the ANME-2a MAGs (Fig. 2) (Supplementary Data 1). The oxidation of methane yielding CH_3_-S-COM is catalyzed by the enzyme Mcr, and then, the methyl group is transferred to H_4_SPT (tetrahydrosarcinapterin) by membrane-bound methyltransferase (Mtr) to form CH_3_-H_4_MPT. Under the function of the reversible CO dehydrogenase/acetyl-CoA synthetase (Cdh) complex, acetyl-CoA is produced from CH_3_-H_4_MPT. Moreover, the genes *acs* and *acd*, which encode AMP-forming acetyl-CoA synthetase (Acs) and ADP-forming acetate-CoA ligase (Acd), respectively, were identified in the ANME-2a genome. Acs is widely distributed in all three domains of life and is considered as the predominant enzyme in the acetate to acetyl-CoA reaction^20^. Acd is a novel enzyme in acetate-forming archaea that catalyzes the conversion of acetyl-CoA to acetate and couples this reaction with the synthesis of ATP (acetyl-CoA + ADP + P_i_ → acetate + ATP + CoA). Acd is considered specific to Archaea, although it is also found in a few genomes of bacteria, such as the propionic acid-producing bacteria *Propionibacterium acidipropionici*^21,22^. In addition, the ANME-2a genomes identified in the present study also possess a complete reversal of the CO_2_-dependent methanogenesis pathway, Embden-Meyerhof-Parnas gluconeogenesis pathway and Wood-Ljungdahl pathway. The acetyl-CoA generated in all of the above diverse metabolic reactions can be converted to acetate via Acd, which would allow ANME-2a to produce energy via substrate-level phosphorylation^23^. Furthermore, 28 *acd* genes were identified in metagenomes mainly belonging to ANME-2a, MBG-D, and methanogens. sHowever, only ANME-2a presents as a high abundance (55.2–80.0%) and has been known as methane oxidizer, thus to be expected as the major player to convert methane into acetate in this simulated cold seep ecosystem (Supplementary Data 2).

The relationship between ANME and bacteria are further interpretated from the metagenomic data. Unlike in ANME-2d or ANME-1, *ech* or *mvh* gene, the key genes to convert energy from hydrogen, have not been identified in ANME-2a MAGs even though the completenesses are over 99%. Therefore it is unlikely that hydrogen is served as electron shuttle between ANME-2a and SRB. Meanwhile, *fsr* gene encoding coenzyme F420-dependent dissimilatory sulfite reductase has been identified, suggesting the possibility of ANME-2a oxidizing methane using sulfite as electron acceptor without a partner. Moreover, MAGs belonging to Desulfobacterales, Desulfitobacteriales, and Burkholderiales possess genes involved in acetate utilizing pathway and sulfate/thiosulfate reduction pathway in their genomes, which makes them potential candidate partners of ANME-2a (Supplementary Data 3).

### Enzyme activities of Acd and Acs

**Fig. 3:**
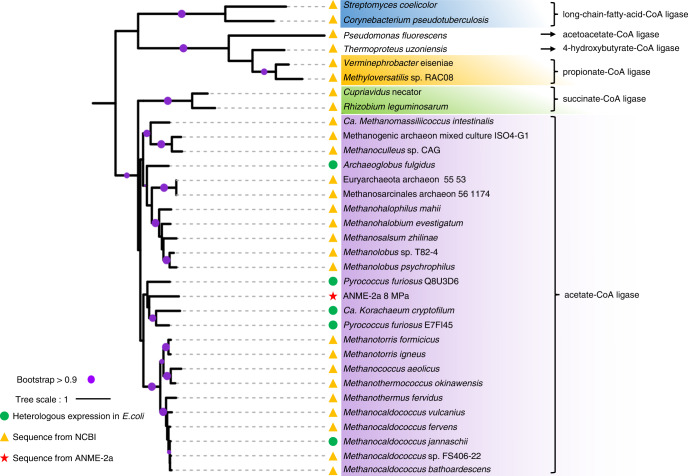
Verification of the *acd* gene sequence from ANME-2a. Sequence from ANME-2a is marked as red asterisk, seqences from NCBI database that have been heterologous expressed in *E.coli* are marked as green dots, and other sequences from NCBI database are marked as yellow triangle. The purple dot indicates the clustering with bootstrap value above 0.9.

**Fig. 4:**
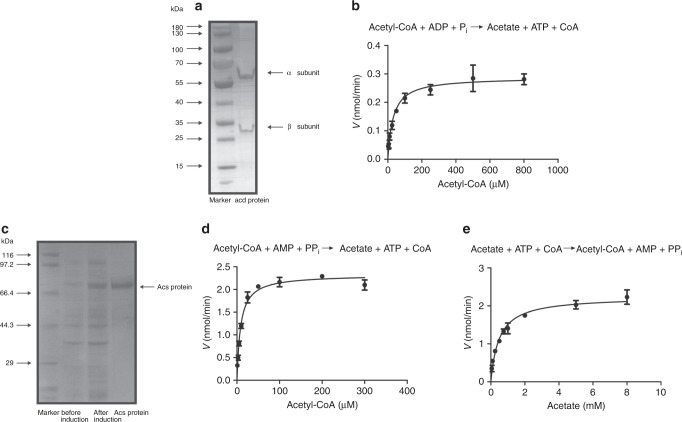
Enzyme purification and activity assay. Purified ADP-forming acetate-CoA ligase (**a**) and acetyl-CoA synthetase (**c**) from ANME-2a as analyzed by SDS-PAGE as well as their activity assay (**b**, **d**, **e**). The catalytic activity is shown as mean value with standard deviation (*n* = 3).

To further verify the metabolic capacity of ANME-2a to produce acetate, we performed heterologous gene expression and activity assays on the two key enzymes Acd and Acs at the final step of acetate formation. Gene sequences were extracted from Bin-8 MPa, and their annotations were verified by constructing phylogenetic trees using IQ-TREE (Fig. 3). The Acd sequence identified in this ANME-2a genome was clustered with those of *Pyrococcus* sp., which have been verified by heterologous gene expression in previous research^24–26^. For Acd, the genes coding for the alpha and beta subunits of Acd were synthesized and subsequently cloned into the pET-28a protein expression vector between the sites NdeI/BamHI and BamHI/SalI, respectively, and an RBS sequence was introduced upstream of the beta subunit. *Escherichia coli* BL21 (DE3) -groEL was used as the expression strain. SDS-PAGE revealed an alpha subunit and beta subunit with apparent molecular masses of 55 kDa and 25 kDa, respectively (Fig. 4a). Detailed information about the protein expression and enzyme activity detection are described in the “Methods” section. The purified enzyme showed catalytic activity for acetate formation. The affinity to acetyl-CoA was *K*_m_ = 31.28 μM (Fig. 4b), similar to the *K*_m_ = 37 μM from *Methanocaldococcus jannaschii* Acd^23^. *M. jannaschii* is a thermophilic methanogenic archaeon using CO_2_ and H_2_ as primary energy sources and can de novo synthetize acetate and pyruvate from CO_2_^27^. We also cloned and expressed the ANME-2a *acs* gene in *Escherichia coli*. The gene coding for Acs was synthesized and subsequently cloned into the vector pET-28a between the NdeI and BamHI sites. The enzyme was overexpressed in Rosetta (DE3) and obtained by protein purification with a molecular mass of 70 kDa (Fig. 4c). Normally, Acs is considered as the key enzyme for acetyl-CoA formation from acetate, CoA and ATP (acetate + ATP + CoA →acetyl-CoA + AMP + PP_i_), and the *K*_m_ values for acetyl-CoA are 2-fold higher than those of acetate^28,29^. However, the Acs purified in this study revealed a higher affinity for acetyl-CoA (*K*_m_ = 8.5 μM) than for acetate (*K*_m_ = 0.49 mM) (Fig. 4d, e), which indicates a preference for acetate production in this case. The enzyme assays of Acd and Acs provide strong evidence to conclude that the conversion from acetyl-CoA to acetate is favorable for ANME-2a.

### Wide distribution of the *acd* gene in cold seep archaea

**Fig. 5:**
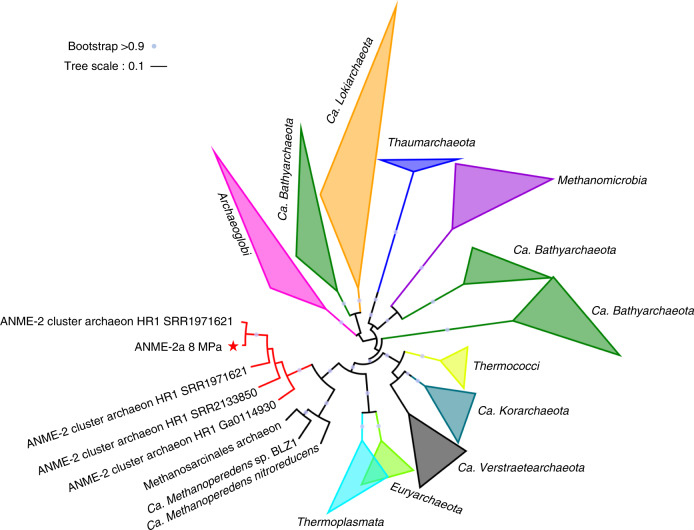
Distribution of *acd* genes among archaea from cold seeps. A total of 113 *acd* genes are identified from 77 metagenomic datasets of cold seep samples. An uncollapsed tree is shown in Supplementary Fig. 3. *Acd* gene from ANME-2a is marked as red asterisk.

To explore the distribution of archaeal acetogenesis in cold seep environments, a phylogenetic tree of *acd* genes from a total of 77 cold seep environments was constructed (Supplementary Fig. 3). A collapsed version of this phylogenetic tree was built to highlight the main taxa (Fig. 5). Among all the ANME and methanogens, only ANME-2, Methanosarcinales, and *Ca*. Verstraetearchaeota contained *acd* genes. In particular, the ANME-2 from methane seepages and subseafloor sediment formed a separate cluster. No ANME-1 or ANME-3 were found to possess the *acd* gene in these metagenomes. Meanwhile, *acd* genes were identified in Archaeoglobi, *Ca*.Bathyarchaeota, Crenarchaeota, Thermococci, and Thermoplasmata (including MBG-D), indicating a wide distribution of acetate metabolism among cold seep archaea.

## Discussion

In this research, a simplified and stable ecosystem supported solely by the AOM process was successfully maintained using a high-pressure continuous bioreactor, allowing us to precisely monitor the ecological dynamics therein. Because of the low solubility of methane at ambient pressure and the extremely low affinity of AOM process for methane (*K*_m_ of 37 mM), high-pressure bioreactors have been applied in AOM studies and obtained higher AOM activity^19^. The high-pressure continuous bioreactor applied in this experiment can control the methane partial pressure and incubation pressure to simulate cold seep eruption at different depths. Furthermore, the continuous flow can remove accumulated metabolites such as CO_2_ and sulfide in a timely manner, alleviating their inhibition of AOM activity and avoiding the toxic effect of sulfide on sulfate-reducing bacteria^30^. The stable and relatively high AOM activities observed during a one-year incubation demonstrated a healthy AOM-supported ecosystem with a microbial community structure similar to those found in nature. For example, the communities retrieved from 23 globally distributed methane seeps were dominated by mainly methane-oxidizing archaea (e.g., ANME-2a/b: ~0–50%) and heterotrophic bacteria (e.g., Gammaproteobacteria: ~0–30%; Alphaproteobacteria, ~0–5%; Clostridia: ~0–50%)^31^, to which high percentages of species in our enriched community were attributed (Fig. 1b). Meanwhile, all seeps harbor extremely diverse bacterial communities including dozens of groups, and approximately 70% of the bacterial community is heterotrophic^31^. The quick consumption of acetate by the heterotrophic population may keep the acetate concentration low, thus the Gibbs free energy to form acetate from methane and bicarbonate is negative, e.g., under our cultivation condition is down to −4.6 kJ/mol. This makes the acetogenesis process energetically beneficial to ANME-2a cells, although it is insufficient to sustain microbial life. AOM process remains as the major energy source to ANME-2a.

The genomic and enzymatic evidence provided by this study of acetate production by ANME-2a could explain the existence of the large heterotrophic communities in cold seep ecosystems, as acetate is a popular carbon source for marine ecosystems. In surface water, acetate has been identified not only as an energy source but also as an important carbon source incorporated into biomass (accounting for 58% of total uptake) by heterotrophic bacteria^32^. In anoxic sediments, acetate was found to be utilized primarily by non-methanogenic heterotrophs as an energy and carbon source, and approximately 10–76% was assimilated into biomass for cell growth^10,33^. The archaeal acetate-producing enzyme Acd represents the major energy-conserving reaction during the fermentation of sugar, peptides, and pyruvate to acetate^34^. Because ANME-2a is phylogenetically related to the acetotrophic methanogen group Methanosarcinales and performs the AOM process via a reverse methanogenesis pathway, and *acd* gene has been identified in ANME-2a and ANME-2d MAGs^17,35,36^, it is reasonable to expect the intracellular conversion of methane into acetate by ANME-2. In addition to acetate, Wegener et al. proposed that during the AOM process, some methylated compounds (i.e., methanol, methylamines, and methyl sulfides) may leak from ANME and thus support the growth of methanogens^37^. The production of organic compounds by ANME may be the result of unbalanced enzymatic activities in the reverse methanogenesis pathway. For example, under the scenario that the production of CH_3_-SCoM and CH_3_-H_4_MPT is faster than their consumption, the generation of acetate is easily achieved. As recently reported, ANME-2d produces acetate indirectly from methane, but via intracellular storage compound, when nitrate or nitrite was supplied as an electron acceptor under a rate-limiting condition^38^. In the natural seeping environments in deep sea, the dissolved methane concentration is much higher than the sulfate concentration, where the electron donor and acceptor have been always imbalanced. Therefore it is likely to be a common phenomenon that the acetate is produced from methane in cold seep ecosystems. Moreover, acetate and other organic compounds have long been proposed as candidate electron shuttles between ANME-2 and SRB to complete the AOM-SR process^39,40^. If such carbon leakage occurs and fuels other heterotrophs, we may expect that the sulfate reduction rates would be lower than the methane oxidation rates and that SRB would grow more slowly than ANME; these expectations have been verified by our results and previous reports^41,42^.

Considering the possibility of producing organic carbon during the AOM process, methane consumption through the AOM process may be underestimated. In our high-pressure continuous incubation experiment, when optimal AOM activity was observed, approximately 1.7 × 10^7^ cellular growth (~0.85 μg carbon) per day were observed (Fig. 1b). Assuming 70% of the cells are heterotrophs and 10–76% of consumed acetate is channeled into biosynthesis according to the previous reports, they would need 0.78–5.95 μg organic carbon per day^6,10,33^. Assuming all the organic carbon originated from methane, and considering approximately 24 μg methane-carbon consumption per day (calculated from the AOM activity in Fig. 1a), 3-25% of the total consumed methane is converted to acetate or other organic compounds that have been previously neglected. Based on the previous calculation, the AOM process consumes up to 300 Tg methane/year, equivalent to ~88% of the methane released from subsurface reservoirs^13^. Considering the missing step of methane conversion to acetate, methane consumption via AOM could be even greater. Because methane is one of the most powerful greenhouse gases, revisiting the methane budget of cold seeps, especially the shallow seeps, where methane is often emitted directly into the atmosphere, is highly significant in terms of predicting global climate change^43^.

**Fig. 6:**
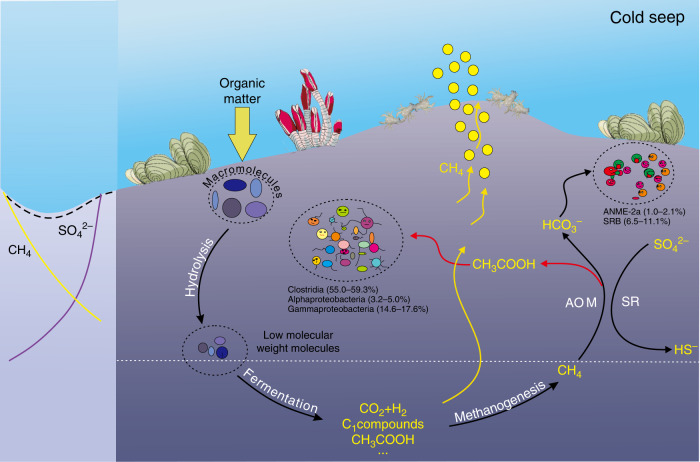
Proposed conceptual diagram of carbon cycling in cold seeps. A proposed conceptual model of carbon cycling in cold seeps: during the classic anaerobic oxidation of methane process, methane can be converted into organic carbon, such as acetate, which further fuels the heterotrophic community in the ecosystem.

In conclusion, cold seep environments host abundant and diverse microbial communities, and nearly 70% of the bacteria are heterotrophs. Metabolic reconstruction of ANME-2a and heterologous expression and activity assays of the ADP-forming acetate-CoA ligase gene *acd* from ANME-2a demonstrate the capability of ANME-2a to produce acetate during methane oxidation. Based on the diversity and metagenomic analysis, the acetate can be supporting a large number of heterotrophic bacteria besides sulfate reducers, such as Firmicutes, Chloroflexi, Actinobacteria, Atribacteria, and Bacteroidetes. Based on these results, we propose a conceptual model of carbon cycling in cold seeps, where acetate release from AOM process is considered (Fig. 6). Our findings expand the metabolic repertoire of ANME-2a and increase understanding of the carbon cycle in cold seep ecosystems.

## Methods

### Continuous high-pressure incubation and activity analysis

The inoculum was originally from Captain Aryutinov Mud Volcano (35° 39.700′N; 07° 20.012′W) at a water depth of 1200 m and had been incubated in a continuous high-pressure bioreactor for years before ANME-2a enrichment was obtained^19,44^. In this study, the methane partial pressure and the incubation pressure were independently controlled in a flow-through high-pressure system^45^. Suggested by the calculated methane affinity for the same ANME-2/SRB community, 8 MPa of methane was provided in the system to achieve the maximum AOM rate^40^. The incubation pressures were further set as 8, 15, and 30 MPa accordingly to mimic the cold seep environments at different water depths and to create dynamics to the microbial community inside the reactor without changing the substrate concentrations. Every liter of medium consisted of the following: NaCl 26 g, MgCl_2_·6H_2_O 5 g, CaCl_2_·2H_2_O 1.4 g, Na_2_SO_4_ 1.3 g, NH_4_Cl 0.3 g, KH_2_PO_4_ 0.1 g, KCl 0.5 g, a bicarbonate solution 30 mL, a vitamin mixture solution 1 mL, a trace element solution 1 mL, a thiamine solution 1 mL, and a vitamin B_12_ solution 1 mL. The bicarbonate solution, the vitamin solutions, and the trace element solution were made according to Widdel and Bak^46^. The pH of the medium was adjusted to 6.8 by adding sulfuric acid. The medium was prepared under a nitrogen atmosphere; it was first saturated with high-pressure methane and then transferred to the bioreactor at a flow rate of 0.1 mL/min. Incubation was performed at 15 °C, and the methane pressure and the incubation pressure were changed every 2 months. To monitor AOM-SR activity, the consumption of methane and sulfate, as well as the production of carbon dioxide and sulfide, were analyzed as previously described^19^. Since we were running a flow through system, the sulfide production rate was calculated by measuring the sulfide concentrations from the inlet and outlet of the incubation vessel, and taking into account of the incubation volume and flow rate. For acetate measurement, 1 mL of sample filtered through a 0.22 μm membrane filter (Merck Millipore, Billerica, MA, USA) was mixed with 0.25 mL 50% H_2_SO_4_, and then 1 mL ether solution containing internal standard 2-methylpentanoic acid (50 μg/mL) was added. Then, the sample was centrifuged at 3,000 rpm for 10 min at 4 °C and incubated at 4 °C for 30 min. The top layer was injected into a Gas Chromatograph-QQQ Mass Spectrometer (GC-QqQ-MS Agilent 7890B-7000D, Agilent Technologies, Santa Clara, CA, USA). The detection limit was lower than 80 nM.

### ^13^C-labeling methane incubation experiment

In order to verify the production of acetate from methane, 20 mL ANME-2a enrichment sample was transferred anaerobically to a 60-mL glass syringe with needle stucked into rubber stopper, and then add 38 mL ^13^C-CH_4_ (Sigma–Aldrich, 99 atom % ^13^C, USA) in the headspace.The sealed needle tubes were then put into the high-pressure vessels (developed in Shanghai Jiao Tong University, China) and pressurized to 8 and 30 MPa, respectively. Considering the dissolvability of methane, the amount of methane we supplied in each syringe is more than sufficient to creat a methane partial pressure up to 8 MPa inside the syringe^41^. Each test had triplication and incubated for 12 months. For ^13^C-labeling acetate measurement, 1 mL of sample filtered through a 0.22 μm membrane filter (Merck Millipore, Billerica, MA, USA) was mixed with 0.2 mL 50% H_2_SO_4_. Afterwards, 1 mL ether solution was added and the sample was vortexed for 1 min. Then, the sample was centrifuged at 12,000 rpm for 20 min at 4 °C and incubated at 4 °C for 30 min. The top layer was injected into a 7890B-7000D GC-QQQ-MS spectrometer (Agilent Technologies, Santa Clara, CA, USA) for analysing. The detection limit was lower than 0.2 μM. Detecting the ratio of ^13^CO_2_/^12^CO_2_ was performed by Gasbench II on-line gas preparation and introduction system (Thermo Fisher Scientific, Bremen, Germany), coupled with 253 Plus isotope ratio mass spectrometer (Thermo Fisher Scientific, Bremen, Germany).

### DNA extraction and 16S rRNA gene analysis

DNA was extracted and purified according to the modified SDS-based method described by Natarjan et al.^47^. Purified DNA was dissolved in 60 μL of ddH_2_O and stored at −80 °C until use. The V4 region of bacterial 16S rRNA genes was amplified by polymerase chain reaction (PCR) with the primer pair 533F (5′-TGCCAGCAGCCGCGGTAA-3′) and Bact806R (5′-GGACTACCAGGGTATCTAATCCTGTT-3′), while the V4–V5 region of archaeal 16S rRNA genes was amplified with the primer pair Arch516F (5′-TGYCAGCCGCCGCGGTAAHACCVGC-3′) and Arch855R (5′-TCCCCCGCCAATTCCTTTAA-3′)^48^. The 50 μL amplification mixture contained 1 μL of each forward and reverse primer, 1 μL of template DNA, 5 μL of 10× Ex Taq buffer, 5 μL of 2.5 mM dNTP mix, 0.25 μL of Ex Taq polymerase (TaKaRa, Tokyo, Japan) and 39.75 μL of ddH_2_O. The PCR conditions for archaeal 16S rRNA gene amplification was as follows: 94 °C for 5 min, followed by 35 cycles at 94 °C for 40 s, 60 °C for 40 s, and 72 °C for 50 s and a final extension at 72 °C for 10 min. For bacteria, the PCR condition was 94 °C for 5 min; 25 cycles of 40 s at 94 °C, 40 s at 58 °C, and 30 s at 72 °C; and a final extension for 10 min at 72 °C. The PCR products were purified with an E.Z.N.A. Gel Extraction Kit (Omega Bio-Tek, Norcross, GA, USA). The 16S rRNA gene amplicons containing the unique barcodes used for each sample were pooled at equal concentrations and sequenced on an Illumina MiSeq platform using 2 × 250-bp cycles at Shanghai Personal Biotechnology Co., Ltd. (Shanghai, China). The raw sequence reads from all the samples were quality-filtered using an average quality value of 20 during demultiplexing; sequences with mean quality score <20 and length <150 bp or any ambiguities were excluded from the analysis. Raw tags were then generated by merging paired-end (PE) reads with FLASH (version 1.2.7)^49^. Further analysis of the 16S rRNA sequence data was performed using the QIIME (version 1.9.1)^50^ software pipeline. The chimeric sequences obtained during the PCR process were removed using VSEARCH^51^. The QIIME-compatible version of the SILVA SSU132 database^52^ was used to identify OTUs with 97% similarity and to assign taxonomy.

### Population quantification

The bacterial and archaeal populations were quantified using qPCR. qPCR amplification was performed using a StepOnePlus Real-Time PCR System (Applied Biosystems, Foster, CA, USA), and all the reactions for bacterial 16S rRNA gene were conducted using SYBR Premix Ex Taq (TaKaRa, Tokyo, Japan). The primer pair bac331F (5′-TCCTACGGGAGGCAGCAGT-3′) and prokaryotic 797R (5′-GGACTACCAGGGTATCTAATCCTGTT-3′) was used for bacteria, and Uni519F (5′-GCMGCCGCGGTAA-3′) and Arc908R (5′-CCCGCCAATTCCTTTAAGTT-3′)^53^ were used for archaea. Each reaction was conducted in triplicate. The qPCR program for bacteria was 50 °C for 2 min, 95 °C for 2 min, and 40 cycles of 95 °C for 15 s and 65 °C for 60 s. For archaea, the program was 95 °C for 15 min, followed by 35 cycles of 95 °C for 30 s, 60 °C for 30 s and 72 °C for 30 s. The quantification standard consisted of a dilution series (between 1 × 10^3^ and 1 × 10^9^ copies/μL) of a known amount of purified PCR product obtained from sediment environmental DNA. The *R*^2^ value for the standard curve was 0.99, and the amplification efficiency was 95–105%. The bacterial cell number was calculated using bacterial 16S rRNA gene copies number divided by 4, which is the mean 16S rRNA operon number of each sample calculated by PICRUSt (Phylogenetic Investigation of Communities by Reconstruction of Unobserved States)^54^. The amount of ANME-2a cells was calculated by using the total archaeal cell number multiplied by its relative abundance in the archaeal community and the SRB cells was calculated by using the total bacterial cell number multiplied by its relative abundance in the bacterial community.

### Metagenomic sequencing and data analysis

For each sample, we constructed one PE DNA library with an insert size of 350 bp following the manufacturer’s instructions (Illumina, San Diego, CA, USA). Sequencing was performed on an Illumina HiSeq X Ten platform using a 2 × 150-bp PE strategy at BGI Co., Ltd. (Shenzhen, China). The metagenomic reads from raw shotgun sequencing were trimmed with sickle (v1.33, http://github.com/najoshi/sickle) using the “pe” option and the default settings. The DNA reads were assembled using a modified IDBA-UD (v1.1.1)^55^ with the following parameters: --step 4, --min_contig 500, --pre_correction, --seed_kmer 55, --maxk 124, --mink 24. Then, the sequencing reads were mapped to the assembled scaffolds using Bowtie (v2.2.8)^56^. After that, the coverage of the assembled scaffolds was calculated by SAMtools (v1.3.1)^57^ and the script from MetaBAT (v2.12.1)^58^. Binning of the assembled metagenomic sequences was performed by both MetaBAT (v2.12.1)^58^ and MyCC^59^ with the meta model. The minimum scaffold lengths were 2.5 Kb for MetaBAT and 1 Kb for MyCC^59^. The quality and rough taxonomy were evaluated by CheckM (v1.0.9)^60^. Manual correction was performed with mmgenome^61^ according to coverage and GC content. The last step was repeated until the completeness was higher than 95% and the contamination was lower than 5%. Protein-coding DNA sequences were determined using Prodigal (v2.6.3)^62^ with the “-meta” setting. Functional information for each predicted protein-coding DNA sequence was collected by a sequence of similarity searches against the KEGG^63^ and COG^64^ databases using DIAMOND^56^ with an *e*-value <1*e*−5.

### Protein expression and purification

The genes coding for the alpha and beta subunits of Acd were synthesized by Shanghai RealGene Biotech using the *Escherichia coli* codon usage. An RBS (ribosome binding site) sequence was introduced upstream of the beta subunit of Acd. The two genes were subsequently cloned into the pET-28a protein expression vector between the sites NdeI/BamHI and BamHI/SalI. Acd protein was heterologously expressed in *E. coli* BL21 (DE3) -groEL strain which was generally provided by Dr. Luying Xun of Washington State University. The expression was induced with 0.2 mM isopropyl-β-d-thiogalactoside in a total volume of 1.0 L at 20 °C for 20 h when the OD_600nm_ reached 0.5. The gene coding for Acs was synthesized by Shanghai RealGene Biotech and subsequently cloned into the vector pET-28a between the sites NdeI and BamHI using the *E. coli* codon usage. Rosetta (DE3) was used as the expression strain which was obtained from TransGen Biotech. The overexpression of Acs in the *E. coli* strain Rosetta (DE3) was indued with 0.2 mM isopropyl-β-d-thiogalactoside in a total volume of 500 mL when the OD_600nm_ reached 0.5. The cells were harvested by centrifugation at 5000 rpm for 5 min and then lysed by sonication in lysis buffer (20 mM Tris-HCl, pH 8.0, 300 mM NaCl, 10% glycerol). The cell lysate was centrifuged at 10,000 rpm for 50 min to remove debris, after which the supernatant was collected and applied to a Ni-NTA column. After washing with a buffer (20 mM Tris-HCl, pH 8.0, 300 mM NaCl, 40 mM imidazole, 10% glycerol), the fusion protein was eluted with another buffer (20 mM Tris-HCl, pH 8.0, 300 mM NaCl, 300 mM imidazole and 10% glycerol) and then dialyzed into storage buffer (20 mM Tris-HCl, pH 8.0, 300 mM NaCl, 50% glycerol). An empty pET-28a vector was used as a control. All chemicals were reagent grade and were purchased from Sigma–Aldrich (St. Louis, MO, USA).

### Enzyme assay

Acs activity was measured in the direction of acetate formation by following PPi-dependent and AMP-dependent HSCoA release from acetyl-CoA with the thiol reagent 5′5-dithiobis (2-nitrobenzoic acid) (DTNB) and measuring the formation of thiophenolate anions at 412 nm (£_412_ = 13.6 mM^−1^ cm^−1^)^65^. The standard mixture contained 20 mM Tris-HCl, pH 8.0, 1.25 M KCl, 1 mM MgCl_2_, 0.1 mM DTNB, 0.05 mM acetyl-CoA, 2 mM AMP, and 2 mM PPi by a previously modified procedure^28^. For the reverse reaction in the acetate decomposition direction, the activity was determined by coupling the reaction with malate dehydrogenase (Mdh) and citrate synthase (Cs) and monitoring the formation of NADH^66^. Acd activity was also determined in the direction of acetate formation by following the ADP-dependent release of coenzyme A (HSCoA) from acetyl-CoA using the thiol reagent DTNB^65^. The assay mixture contained 20 mM Tris-HCl, pH 8.0, 40 mM K_3_PO_4_, 1 mM MgCl_2_, 0.1 mM DTNB, 0.05 mM acetyl-CoA, 2 mM ADP by a previously modified procedure^67^. The reaction was started by the addition of the enzyme, and the formation of the thiophenolate anion was measured at 412 nm (£_412_ = 13.6 mM^−1^ cm^−1^).

### Phylogenetic tree construction

To verify the *acd* gene sequence from ANME-2 MAG, *acd* gene expressed in *E. coli* from the NCBI database and the sequence in MAG 8 MPa were aligned with MAFFT-linsi (v7.313)^68^ and trimmed with trimAL (v1.4)^69^ using “automated1”. The final alignments has 513 trimmed columns. A phylogenetic tree was constructed by IQ-TREE (v1.6.6)^70^ with “LG + G4” model and 1,000 ultrafast bootstraps. To examine the distribution of *acd* genes among archaea from cold seeps, 77 cold seep metagenomic datasets were collected. If the dataset did not have available annotations in the IMG database, then its raw reads were trimmed with BBMap (v36.27)^71^. A total of 113 *acd* genes were identified by eggNOG-mapper^72^ against the eggNOG database from 77 cold seep metagenomic datasets (accession numbers were shown in Supplementary Data 4). Contigs were assembled by Megahit (v1.0.6-hotfix1)^73^, and ORFs were called by Prodigal. And the taxonomy of each gene is determined by nr database with DIAMOND^56^. Next, the alignment with 177 columns was used to construct a phylogenetic tree with the same method as above except with LG + I + G4 models and 1,000 ultrafast bootstraps (Supplementary Fig. 3). The tree of ANME-2 MAGs was constructed with a 700 amino acids-long concatenation of 122 archaeal marker genes (Supplementary Fig. 2). Mafft-linsi was used for alignment for each gene set, and we have removed the positions with more gaps than 50% of the actual amino acid sequences. The tree was inferred by IQ-TREE (v1.6.6)^70^ with LG + G4 model and 1,000 ultrafast bootstraps.

### Taxonomy assignment of *acd* genes and their abundance by TPM

According to the KEGG result, *acd* (K01905) genes were extracted from the whole metagenome protein-coding sequence of 8, 15, 30, 8 MPa (II). Their taxonomy information was determined by the best hit against NCBI non-redundant database using DIAMOND (v0.9.24)^56^ with an *e*-value <1*e*−5 and abundance was evaluated by transcripts per million (TPM), which is a normalization method based on gene length and corresponding mapped reads number. Firstly featureCounts (v1.6.3)^74^ was used to count how many reads were mapped to each gene. Then TPM was calculated by a custom python script.

### MAGs with sulfur and acetate metabolism potential

To obtain metagenomic-assembled genome (MAG), three programs were performed individually, MetaBat2 (v2.12.1)^58^, MaxBin2 (v2.2.6)^75^ with “--markerset 40” and CONCOCT (v1.0.0)^76^. Then DAS Tool (v1.1.1)^77^ was run to integrate these three sets of MAGs with “--search_engine diamond --score_threshold 0”. Completeness and contamination were evaluated by CheckM (v1.1.12)^60^ and taxonomy were determined by GTDB-Tk (v1.0.2)^78^. Based on these, MAGs with high quality (completeness > 90%, contamination < 5%), which has the potential of sulfur and acetate metabolism, were found in sample 8 and 8 MPa (II) (Supplementary Data 3). Firstly, we calculated the relative abundance of each MAG in their metagenomic datasets, respectively. It was the percentage of mapped quality-controlled metagenome short reads of each MAG in all mapped quality-controlled reads using BBMap (v36.27)^71^. Then the abundance of the corresponding OTU in QIIME result was also listed, if a MAG could be classified to genus level. Three MAGs have no taxonomy information in GTDB-Tk, suggesting a novel clade. That made trouble connecting them with OTU QIIME result because of the inconsistence between GTDB database^79^ and SILVA SSU132 database^52^, which are used as a classifier reference for QIIME (version 1.9.1)^50^.

## Supplementary information

Supplementary Information

Description of Additional Supplementary Files

Supplementary Data 1

Supplementary Data 2

Supplementary Data 3

Supplementary Data 4

## Data Availability

The 16S rRNA gene amplicon reads have been deposited in the NCBI (National Center for Biotechnology Information) Sequence Read Archive database under the accession numbers SRR10337033 (Initial), SRR10337032 (8 MPa), SRR10337031 (15 MPa), SRR10337030 (30 MPa), and SRR10337029 (8 MPa II), respectively. The metagenome-assembled genomes from the current study have been deposited in the NODE (the National Omics Data Encyclopedia) database under the project number OEP000824. All other data are available in the paper or the Supplementary Information. Source data are provided with this paper.

## Source data

Source Data

## References

[1] Luff R, Wallmann K (2003). Fluid flow, methane fluxes, carbonate precipitation and biogeochemical turnover in gas hydrate-bearing sediments at Hydrate Ridge, Cascadia Margin: numerical modeling and mass balances. Geochim. et. Cosmochim. Acta.

[2] Brown K (2005). Correlated transient fluid pulsing and seismic tremor in the Costa Rica subduction zone. Earth Planet. Sci. Lett..

[3] Ruff SE (2013). Microbial communities of deep-sea methane seeps at Hikurangi continental margin (New Zealand). PLoS ONE.

[4] Felden J (2014). Anaerobic methanotrophic community of a 5346-m-deep vesicomyid clam colony in the Japan Trench. Geobiology.

[5] Pop Ristova P (2012). Bacterial diversity and biogeochemistry of different chemosynthetic habitats of the REGAB cold seep (West African margin, 3160 m water depth). Biogeosciences.

[6] Knittel K, Boetius A (2009). Anaerobic oxidation of methane: progress with an unknown process. Annu. Rev. Microbiol..

[7] Niemann H (2006). Novel microbial communities of the Haakon Mosby mud volcano and their role as a methane sink. Nature.

[8] Schreiber L (2010). Identification of the dominant sulfate-reducing bacterial partner of anaerobic methanotrophs of the ANME-2 clade. Environ. Microbiol..

[9] Holler T (2012). Carbon and sulfur back flux during anaerobic microbial oxidation of methane and coupled sulfate reduction. Proc. Natl Acad. Sci..

[10] Michaelis W (2002). Microbial reefs in the black sea fueled by anaerobic oxidation of methane. Science.

[11] Treude T (2007). Consumption of methane and CO2 by methanotrophic microbial mats from gas seeps of the anoxic black sea. Appl. Environ. Microbiol..

[12] Jørgensen BB, Boetius A (2007). Feast and famine—microbial life in the deep-sea bed. Nat. Rev. Microbiol..

[13] Reeburgh WS (2007). Oceanic methane biogeochemistry. Chem. Rev..

[14] Lee J-W (2013). Microbial community structures of methane hydrate-bearing sediments in the Ulleung Basin, East Sea of Korea. Mar. Pet. Geol..

[15] Lee YM (2018). Genomic insight into the predominance of candidate phylum atribacteria JS1 lineage in marine sediments. Front. Microbiol..

[16] Cui H (2019). Microbial diversity of two cold seep systems in gas hydrate-bearing sediments in the South China Sea. Mar. Environ. Res..

[17] Wang F-P (2013). Methanotrophic archaea possessing diverging methane-oxidizing and electron-transporting pathways. ISME J..

[18] Soo VWC (2016). Reversing methanogenesis to capture methane for liquid biofuel precursors. Microb. Cell Factor..

[19] Zhang Y, Henriet J-P, Bursens J, Boon N (2010). Stimulation of in vitro anaerobic oxidation of methane rate in a continuous high-pressure bioreactor. Bioresour. Technol..

[20] Ingram-Smith C, Woods BI, Smith KS (2006). Characterization of the acyl substrate binding pocket of acetyl-CoA synthetase. Biochemistry.

[21] Schmidt M, Schönheit P (2013). Acetate formation in the photoheterotrophic bacterium Chloroflexus aurantiacus involves an archaeal type ADP-forming acetyl-CoA synthetase isoenzyme I. FEMS Microbiol. Lett..

[22] Parizzi LP (2012). The genome sequence of Propionibacterium acidipropionici provides insights into its biotechnological and industrial potential. BMC Genomics.

[23] Musfeldt M, Schonheit P (2002). Novel type of ADP-forming acetyl coenzyme A synthetase in hyperthermophilic archaea: heterologous expression and characterization of isoenzymes from the sulfate reducer Archaeoglobus fulgidus and the methanogen Methanococcus jannaschii. J. Bacteriol..

[24] Mai X, Adams MW (1996). Purification and characterization of two reversible and ADP-dependent acetyl coenzyme A synthetases from the hyperthermophilic archaeon Pyrococcus furiosus. J. Bacteriol..

[25] Glasemacher J, Bock AK, Schmid R, Schonheit P (1997). Purification and properties of acetyl-CoA synthetase (ADP-forming), an archaeal enzyme of acetate formation and ATP synthesis, from the hyperthermophile Pyrococcus furiosus. Eur. J. Biochem..

[26] Musfeldt M, Selig M, Schonheit P (1999). Acetyl coenzyme A synthetase (ADP forming) from the hyperthermophilic Archaeon pyrococcus furiosus: identification, cloning, separate expression of the encoding genes, acdAI and acdBI, in Escherichia coli, and in vitro reconstitution of the active heterotetrameric enzyme from its recombinant subunits. J. Bacteriol..

[27] Jones WJ (1983). Methanococcus jannaschii sp. nov., an extremely thermophilic methanogen from a submarine hydrothermal vent. Arch. Microbiol..

[28] Bräsen C, Schönheit P (2005). AMP-forming acetyl-CoA synthetase from the extremely halophilic archaeon Haloarcula marismortui: purification, identification and expression of the encoding gene, and phylogenetic affiliation. Extremophiles.

[29] Bräsen C, Urbanke C, Schönheit P (2004). A novel octameric AMP-forming acetyl-CoA synthetase from the hyperthermophilic crenarchaeon Pyrobaculum aerophilum. FEBS Lett..

[30] Rabus R, Heider J (1998). Initial reactions of anaerobic metabolism of alkylbenzenes in denitrifying and sulfate-reducing bacteria. Arch. Microbiol..

[31] Ruff SE (2015). Global dispersion and local diversification of the methane seep microbiome. Proc. Natl Acad. Sci..

[32] Zhuang G-C (2019). Significance of acetate as a microbial carbon and energy source in the water column of Gulf of Mexico: implications for marine carbon cycling. Glob. Biogeochem. Cycles.

[33] Zhuang G-C, Montgomery A, Joye SB (2019). Heterotrophic metabolism of C1 and C2 low molecular weight compounds in northern Gulf of Mexico sediments: controlling factors and implications for organic carbon degradation. Geochim. et. Cosmochim. Acta.

[34] Bräsen C, Schmidt M, Grötzinger J, Schönheit P (2008). Reaction mechanism and structural model of ADP-forming acetyl-CoA synthetase from the hyperthermophilic archaeon Pyrococcus furiosus. J. Biol. Chem..

[35] Haroon MF (2013). Anaerobic oxidation of methane coupled to nitrate reduction in a novel archaeal lineage. Nature.

[36] Ino K (2017). Ecological and genomic profiling of anaerobic methane-oxidizing archaea in a deep granitic environment. ISME J..

[37] Wegener G (2016). Metabolic capabilities of microorganisms involved in and associated with the anaerobic oxidation of methane. Front. Microbiol..

[38] Cai C (2019). Acetate production from anaerobic oxidation of methane via intracellular storage compounds. Environ. Sci. Technol..

[39] Valentine DL, Reeburgh WS (2000). New perspectives on anaerobic methane oxidation. Environ. Microbiol..

[40] Stams AJM, Plugge CM (2009). Electron transfer in syntrophic communities of anaerobic bacteria and archaea. Nat. Rev. Microbiol..

[41] Deusner C, Meyer V, Ferdelman TG (2010). High-pressure systems for gas-phase free continuous incubation of enriched marine microbial communities performing anaerobic oxidation of methane. Biotechnol. Bioeng..

[42] Timmers PHA (2015). Growth of anaerobic methane-oxidizing archaea and sulfate-reducing bacteria in a high-pressure membrane capsule bioreactor. Appl. Environ. Microbiol..

[43] Weber T, Wiseman NA, Kock A (2019). Global ocean methane emissions dominated by shallow coastal waters. Nat. Commun..

[44] Zhang Y (2011). Enrichment of a microbial community performing anaerobic oxidation of methane in a continuous high-pressure bioreactor. BMC Microbiol..

[45] Zhang Y, Li X, Bartlett DH, Xiao X (2015). Current developments in marine microbiology: high-pressure biotechnology and the genetic engineering of piezophiles. Curr. Opin. Biotechnol..

[46] Widdel F, Bak F (1992). Gram-negative mesophilic sulfate-reducing bacteria. Prokaryotes.

[47] Natarajan VP (2016). A modified SDS-based DNA extraction method for high quality environmental DNA from seafloor environments. Front. Microbiol..

[48] Klindworth A (2012). Evaluation of general 16S ribosomal RNA gene PCR primers for classical and next-generation sequencing-based diversity studies. Nucleic Acids Res..

[49] Magoc T, Salzberg SL (2011). FLASH: fast length adjustment of short reads to improve genome assemblies. Bioinformatics.

[50] Caporaso JG (2010). QIIME allows analysis of high-throughput community sequencing data. Nat. Publ. Group.

[51] Edgar RC (2011). UCHIME improves sensitivity and speed of chimera detection. Bioinformatics.

[52] Quast C (2013). The SILVA ribosomal RNA gene database project: improved data processing and web-based tools. Nucleic Acids Res..

[53] Jorgensen SL (2012). Correlating microbial community profiles with geochemical data in highly stratified sediments from the Arctic Mid-Ocean Ridge. Proc. Natl Acad. Sci..

[54] Langille MGI (2013). Predictive functional profiling of microbial communities using 16S rRNA marker gene sequences. Nat. Biotechnol..

[55] Peng Y, Leung HCM, Yiu SM, Chin FYL (2012). IDBA-UD: a de novo assembler for single-cell and metagenomic sequencing data with highly uneven depth. Bioinformatics.

[56] Buchfink B, Xie C, Huson DH (2014). Fast and sensitive protein alignment using DIAMOND. Nat. Methods.

[57] Li H (2009). The sequence Alignment/Map format and SAMtools. Bioinformatics.

[58] Kang DD (2019). MetaBAT 2: an adaptive binning algorithm for robust and efficient genome reconstruction from metagenome assemblies. PeerJ.

[59] Lin H-H, Liao Y-C (2016). Accurate binning of metagenomic contigs via automated clustering sequences using information of genomic signatures and marker genes. Sci. Rep..

[60] Parks DH (2015). CheckM: assessing the quality of microbial genomes recovered from isolates, single cells, and metagenomes. Genome Res..

[61] Karst, S. M., Kirkegaard, R. H. & Albertsen, M. mmgenome: a toolbox for reproducible genome extraction from metagenomes. *bioRxiv* 10.1101/059121 (2016).

[62] Hyatt D, Locascio PF, Hauser LJ, Uberbacher EC (2012). Gene and translation initiation site prediction in metagenomic sequences. Bioinformatics.

[63] Kanehisa M, Goto S (2000). KEGG: Kyoto encyclopedia of genes and genomes. Nucleic Acids Res..

[64] Galperin MY, Makarova KS, Wolf YI, Koonin EV (2014). Expanded microbial genome coverage and improved protein family annotation in the COG database. Nucleic Acids Res..

[65] Srere PA, Brazil H, Gonen L (1963). The citrate condensing enzyme of Pigeon breast muscle and moth flight muscle. Acta Chem. Scand..

[66] Castaño-Cerezo S, Bernal V, Cánovas M (2012). Acetyl-coenzyme A synthetase (Acs) assay. Bio-Protocol.

[67] Sánchez LB, Galperin MY, Müller M (2000). Acetyl-CoA synthetase from the Amitochondriate eukaryote giardia lamblia belongs to the newly recognized superfamily of Acyl-CoA synthetases (Nucleoside Diphosphate-forming). J. Biol. Chem..

[68] Katoh K, Misawa K, Kuma KI, Miyata T (2002). MAFFT: a novel method for rapid multiple sequence alignment based on fast Fourier transform. Nucleic Acids Res..

[69] Capella-Gutierrez S, Silla-Martinez JM, Gabaldon T (2009). trimAl: a tool for automated alignment trimming in large-scale phylogenetic analyses. Bioinformatics.

[70] Nguyen L-T, Schmidt HA, Von Haeseler A, Minh BQ (2014). IQ-TREE: a fast and effective stochastic algorithm for estimating maximum-likelihood phylogenies. Mol. Biol. Evol..

[71] Bushnell B (2014). BBMap: A Fast, Accurate, Splice-Aware Aligner..

[72] Huerta-Cepas J (2017). Fast genome-wide functional annotation through orthology assignment by eggNOG-Mapper. Mol. Biol. Evol..

[73] Li D (2016). MEGAHIT v1.0: a fast and scalable metagenome assembler driven by advanced methodologies and community practices. Methods.

[74] Liao Y, Smyth GK, Shi W (2014). featureCounts: an efficient general purpose program for assigning sequence reads to genomic features. Bioinformatics.

[75] Wu Y-W (2014). MaxBin: an automated binning method to recover individual genomes from metagenomes using an expectation-maximization algorithm. Microbiome.

[76] Alneberg J (2014). Binning metagenomic contigs by coverage and composition. Nat. Methods.

[77] Sieber CMK (2018). Recovery of genomes from metagenomes via a dereplication, aggregation and scoring strategy. Nat. Microbiol..

[78] Chaumeil P-A, Mussig AJ, Hugenholtz P, Parks DH (2019). GTDB-Tk: a toolkit to classify genomes with the Genome Taxonomy Database. Bioinformatics.

[79] Parks DH (2018). A standardized bacterial taxonomy based on genome phylogeny substantially revises the tree of life. Nat. Biotechnol..

