# Supplementary material for: Genomic and enzymatic evidence of acetogenesis by anaerobic methanotrophic archaea
Source: Nat Commun. 2020 Aug 7;11:3941. doi: 10.1038/s41467-020-17860-8 (PMC7414198; doi:10.1038/s41467-020-17860-8)
Supplement: Supplementary file 1 — Supplementary Information [file 41467_2020_17860_MOESM1_ESM.pdf]

SUPPLEMENTARY INFORMATION

Genomic and enzymatic evidence of acetogenesis by anaerobic methanotrophic archaea

Yang et al.

Includes:

Supplementary Fig. 1-3

Supplementary Table 1

Supplementary Fig. 1

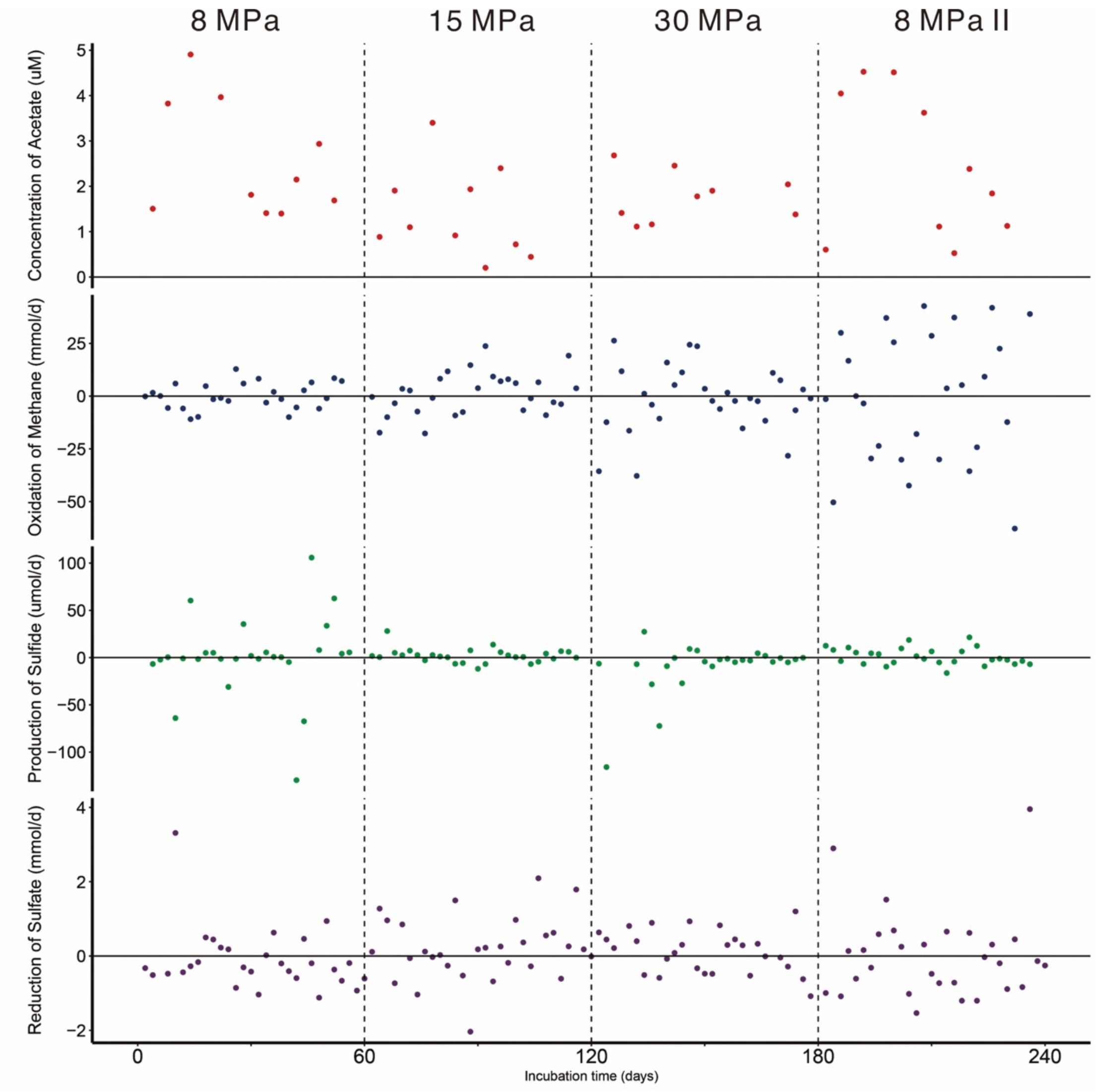

Supplementary Fig. 1. AOM activity tracked by the measurements of the chemical composition during the incubation experiment. The production of sulfide, concentration of acetate, oxidation of methane, reduction of sulfate under different incubation pressures are plotted as dots.

Supplementary Fig. 2

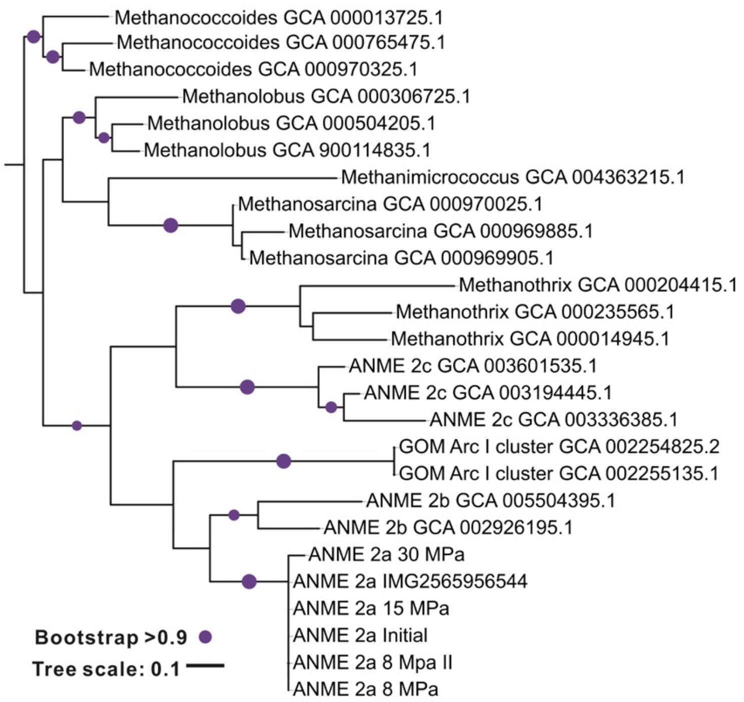

Supplementary Fig. 2. Phylogenetic trees showing the placement of the ANME-2a MAGs (metagenome-assembled genomes). The Tree of ANME-2a MAGs is constructed with a 700 amino acids-long concatenation of 122 archaeal marker genes. Mafft-linsi is used for alignment for each gene set, and we have removed the positions with more gaps than 50% of the actual amino acid sequences. The tree is inferred by IQ-TREE with LG+G4 model and 1,000 ultrafast bootstraps.

Supplementary Fig. 3

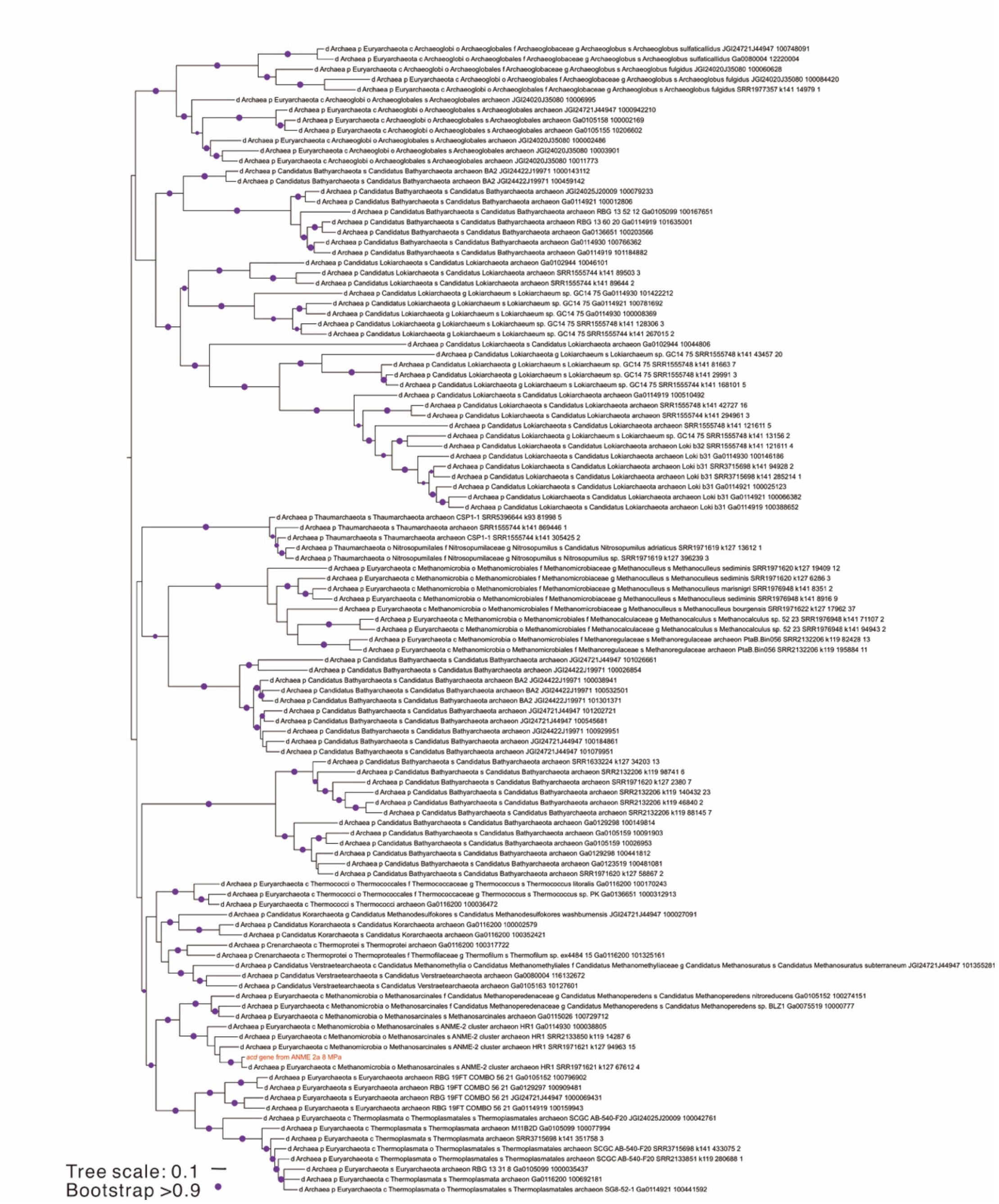

Supplementary Fig. 3. Distribution of *acd* genes among archaea from cold seeps. A phylogenetic tree was used to indicate the distribution of *acd* genes among the archaea from cold seep ecosystems. Total 113 *acd* genes were identified by eggNOG-mapper against the eggNOG database from 77

cold seep metagenomic datasets, and the taxonomy of each gene was determined by NCBI nr database with DIAMOND. The phylogenetic tree was constructed by IQ-TREE (v1.6.6). *Acd* gene from ANME-2a was marked as red.

Supplementary Table

Supplementary Table 1. Overview of ANME-2a metagenome-assembled genomes obtained in this study.

Bin_ID	Completeness (%)	Contamination (%)	G+C (%)	Contigs	N50	Size (M)
Initial	98.69	1.31	43.3	118	50,462	2.9
8 MPa	99.35	1.31	42.9	124	48,079	3.2
15 MPa	96.73	1.47	43.1	124	40,799	3.0
30 MPa	95.03	4.84	44.7	288	11,332	2.6
8 MPa II	99.35	2.61	42.9	133	44,618	3.2
