# Supplementary material for: Genomic and enzymatic evidence of acetogenesis by anaerobic methanotrophic archaea
Source: Nat Commun. 2020 Aug 7;11:3941. doi: 10.1038/s41467-020-17860-8 (PMC7414198; doi:10.1038/s41467-020-17860-8)
Supplement: Supplementary file 2 — Description of Additional Supplementary Files [file 41467_2020_17860_MOESM2_ESM.pdf]

Description of Additional Supplementary Files

File Name: Supplementary Data 1

Description: Genes information of ANME-2a metabolic pathways.

File Name: Supplementary Data 2

Description: Taxonomy assignment of *acd* genes and their abundance based on TPM.

File Name: Supplementary Data 3

Description: Abundance of the MAGs (metagenome-assembled genomes) possessing the sulfur and acetate metabolism potential.

File Name: Supplementary Data 4

Description: List of the data source to analyze the *acd* gene distribution in cold seeps.
